# Suppressing fear in the presence of a safety cue requires infralimbic cortical signaling to central amygdala

**DOI:** 10.1038/s41386-023-01598-0

**Published:** 2023-05-15

**Authors:** Ka Ng, Michael Pollock, Abraham Escobedo, Brent Bachman, Nanami Miyazaki, Edward L. Bartlett, Susan Sangha

**Affiliations:** 1 Department of Psychological Sciences, Purdue University, West Lafayette, IN 47907 USA; 2 Department of Biological Sciences, Purdue University, West Lafayette, IN 47907 USA; 3 Weldon School of Biomedical Engineering, Purdue University, West Lafayette, IN 47907 USA; 4 Department of Psychiatry, Indiana University School of Medicine, Indianapolis, IN 46202 USA

**Keywords:** Classical conditioning, Amygdala

## Abstract

Stressful events can have lasting and impactful effects on behavior, especially by disrupting normal regulation of fear and reward processing. Accurate discrimination among environmental cues predicting threat, safety or reward adaptively guides behavior. Post-traumatic stress disorder (PTSD) represents a condition in which maladaptive fear persists in response to explicit safety-predictive cues that coincide with previously learned threat cues, but without threat being present. Since both the infralimbic cortex (IL) and amygdala have each been shown to be important for fear regulation to safety cues, we tested the necessity of specific IL projections to the basolateral amygdala (BLA) or central amygdala (CeA) during safety recall. Male Long Evans rats were used since prior work showed female Long Evans rats did not acquire the safety discrimination task used in this study. Here, we show the infralimbic projection to the central amygdala was necessary for suppressing fear cue-induced freezing in the presence of a learned safety cue, and the projection to the basolateral amygdala was not. The loss of discriminative fear regulation seen specifically during IL->CeA inhibition is similar to the behavioral disruption seen in PTSD individuals that fail to regulate fear in the presence of a safety cue.

## Introduction

Accurate discrimination of environmental cues predicting safety, fear or reward is important for survival and for initiating the proper emotional response. A loss of stimulus discrimination is seen in disorders such as post-traumatic stress disorder (PTSD), resulting in generalized fear responses to nonthreatening stimuli [1]. Individuals with PTSD also typically do not show adaptive fear regulation when safety cues coincide with fear cues [2]. Since cues signifying safety have the power to modulate both fear and reward-seeking behaviors by informing the organism whether or not the environment is safe, the circuitries governing safety, fear and reward behaviors are intertwined. To test this, we have used a conditional discrimination task in which a reward cue predicts sucrose, a fear cue predicts footshock, and a safety cue coinciding with the fear cue predicts the absence of footshock (fear + safety) [3–9]. Across these studies male rats typically show a significant reduction in freezing levels to the fear + safety cue compared to the fear cue, while female rats typically do not [5].

To test the neural circuits responsible for the fear reducing effects of the safety cue in this conditional discrimination task, we have largely focused on the prefrontal cortex and amygdala, having shown thus far the infralimbic cortex and basolateral amygdala are engaged during the fear + safety cue [3, 7, 9]. More specifically, our recent data has shown a large proportion of individual neurons within the infralimbic cortex (IL) respond with excitation to the fear + safety cue, with the level of excitation being negatively correlated with expressed freezing during the fear + safety cue [3]. That is, excitatory responses in the IL to a fear + safety conflict cue was correlated with better safety behavior. This is in line with our other prior work where inactivating the IL with a mix of muscimol and baclofen prevented safety expression, i.e., fear levels were equally high to the fear cue and the fear + safety cue [7]. These data are consistent with the viewpoint that the IL is engaged during conflicting circumstances where an adaptive behavior needs to be selected over a maladaptive behavior, particularly when this involves behavior based on competing contingencies [10]. In other words, the IL may be promoting adaptive behavior under the conflicting scenario of both a fear and safety cue being presented concurrently, and the adaptive behavior being fear downregulation given the absence of the aversive stimulus.

Studies investigating neural circuits for reducing fear via cued fear extinction have heavily implicated projections from the IL to the amygdala in promoting fear downregulation [11, 12]. Optogenetically manipulating IL signaling to the BLA during either fear extinction acquisition or extinction recall, has shown the critical need for IL input to the BLA during fear extinction acquisition, but not extinction recall [13], to successfully downregulate fear expression. This suggests that expression of previously learned conditional discrimination of fear vs. fear + safety cues would not depend on IL signaling to the BLA, but instead upon another IL target, given that we have previously shown that global IL inactivation prevents safety expression [7]. Here, we tested the hypothesis that IL input to the central amygdala would be critical for expression of learned safety, and that IL input to the basolateral amygdala would not be.

## Materials and methods

### Subjects

Thirty-seven male Long Evans rats (Blue Spruce; Envigo, Indianapolis) weighing 250–275 g upon arrival were single-housed under a 12 h light/dark cycle (lights on 09:00), acclimated to housing conditions for 1 week, and then handled for 1 week before commencing experiments. We have previously shown that female rats do not show fear suppression during the fear + safety cue, but males do [5]. Since the purpose of the current study was to impair fear suppression during the fear + safety cue, only male subjects were used. All procedures were performed during the light cycle and approved by the Purdue Animal Care and Use Committee. Rats had ad libitum access to food and water up until the first training session, at which point they received 20–22 g of food per day after their daily training session for the remainder of the experiment.

### Surgery and virus procedures

Rats were deeply anesthetized with isoflurane and body temperature maintained with an electric heating pad with temperature anal probe to control the level of heat supplied by the pad throughout the surgery. A Cre-dependent AAV (pAAV-hSyn-DIO-hM4D(Gi)-mCherry; Addgene) was micro-injected into the infralimbic cortex of all rats in experiments 2 and 3 (*n* = 19) (IL: AP = +3.0 mm; ML = ±0.5 mm; DV = −5.0 mm), and a Cre-expressing AAV (AAV2(retro)-eSYN-EGFP-T2A-iCre-WPRE; Vector BioLabs) was micro-injected into either the basolateral amygdala (*n* = 8) (BLA: AP = −2.75 mm; ML = ±4.75 mm; DV = −8.25 mm) or central amygdala (*n* = 11) (CeA: AP = −2.75 mm; ML = ±4.25 mm; DV = −8.00 mm). Before injection, 1 ml of the viral stock was drawn up into a 33-gauge bilateral injector needle and connected to a gastight microliter syringe driven by an infusion pump (Harvard Apparatus). After a hole had been drilled into the skull, the microinjector needle was lowered into the brain. A volume of 0.5 µl/hemisphere/brain region of virus was expelled from the pump over 10 min. Each rat had a total of four surgical targets due to two brain regions being targeted bilaterally. The injector needle was left in place for ten additional minutes to allow for diffusion from the site and to prevent reflux. Injectors were then retracted, drill holes filled with sterile bone wax, and the wound closed with monofilament sutures. Animals were allowed to recover from surgery with ad libitum access to food and water for 4 weeks prior to reward conditioning.

### In vivo recordings

Three virus-free control rats and a subset of rats with hM4 expressed within the IL -> BLA projection (*n* = 2) were taken for in vivo recordings within the IL. For one rat, recordings were taken after the completion of behavioral conditioning (8–9 weeks post-AAV injection). For the other rat, behavioral conditioning did not occur but instead recordings were taken at the equivalent time point of DC4/DC5 post-AAV injection (6 weeks). Single-unit recording protocols described here are similar to those used previously [14, 15]. Animals were administered ketamine (VetaKet, 60 mg/kg, i.m.) and dexmedetomidine (Dexdomitor, 0.15 mg/kg, i.m.) to achieve an anesthetic state. Heart rate and blood oxygenation was monitored with a pulse oximeter. Physiological body temperature was maintained with a water-circulating heating pad. Toe pinch reflex was assessed every 30 min. If the reflex was present, supplemental doses of ketamine were administered intramuscularly.

A small craniotomy, ~2 × 2 mm, was centered over IL (AP + 3.20 mm; ML ±0.50 mm) [16]. Single-unit recordings were made in IL using tungsten electrodes (A-M Systems) encased in a glass capillary that was advanced using a hydraulic microdrive (Narishige). The electrode was slowly advanced to 4.6 mm, or just at the approximate depth of IL [16]. The electrode was then advanced until a spontaneously active unit was identified and isolated. Baseline recordings of spontaneous activity were recorded in 22 consecutive 0.5 s blocks. Electrode outputs were sent through a headstage (RA4, Tucker-Davis Technologies, TDT) and amplifier (RA4PA preamplifier, TDT) and recorded (RZ5, TDT) at a sampling rate of 24.414 kHz. Signals were filtered from 500 to 5000 Hz and spike-sorted using OpenEx and RPvdsEx software (TDT). After baseline recordings, CNO was administered (3 mg/kg, i.p.). Recordings of spontaneous activity were collected (22 trials, 0.5 s blocks) every 20 min over the 40–120 min post-injection time window. Recording data were expressed as average spontaneous spikes per trial, averaged across the 22 trials per time point per rat. A two-way ANOVA was used to compare time 0 min, the time point when CNO was injected i.p., to 40, 60, 80, 100 and 120 min post-CNO injection.

### CNO injections

Clozapine-N-oxide (CNO) was obtained from the NIMH Chemical Synthesis and Drug Supply Program. CNO was dissolved in vehicle (95% dH_2_O, 5% DMSO) and rats received a dose of 3 mg/kg of CNO intraperitoneally (Experiment 1: *n* = 16; Experiment 2: *n* = 11; Experiment 3: *n* = 11).

### Apparatus

The training chambers were 12 Med Associates Plexiglas boxes (28 cm length × 21 cm width × 35 cm height) encased in sound-attenuating chambers (Med Associates, ST Albans, VT). 10% liquid sucrose (100 μl) was delivered through a recessed port located in the center of one wall, containing an infrared beam for detecting port entries and exits. There were two lights (28 V, 100 mA), one on each side of the port for delivering the 20 s continuous light cue, and a house light (28 V, 100 mA) located at the top of the wall opposite to the port for providing constant background illumination. Next to the house light was a “tweeter” speaker (ENV-224BM) for delivering auditory cues. Footshocks were delivered through the grid floor by a constant current aversive stimulator (ENV-414S). A side-view video camera located on the door of the sound-attenuating chamber recorded the rat’s behavior for offline video analyses.

### Behavioral conditioning

Three stimuli were used as cues: a 20 s continuous 3 kHz tone (70 dB) served as the reward cue, a 20 s pulsing 11 kHz tone (200 ms on, 200 ms off; 70 dB) as the fear cue, and a 20 s continuous light (28 V, 100 mA) as the safety cue. Stimuli were not counterbalanced in this study but our prior work have shown no differences in learning amongst these stimuli across reward, fear, or safety [3, 5, 9].

**Fig. 1:**
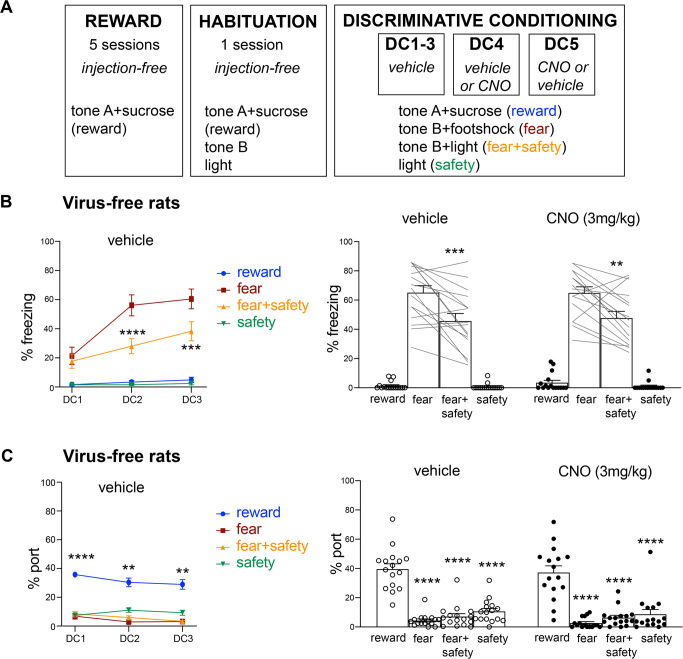
Experiment 1: CNO in virus-free rats did not affect freezing or port behavior. **A** Schematic of behavioral procedure for all rats. Reward training to tone A was paired with sucrose delivery across five sessions. One day later, a habituation session, which still consisted of reward training to tone A, also included pre-exposure trials to tone B and light cue. Discriminative conditioning (DC1-5) continued to deliver tone A-sucrose reward trials. It also paired tone B with footshock (fear cue), presented tone B with light without any footshock (fear + safety cue), and included light alone trials (safety cue). Vehicle injections were administered to all rats 20 min prior to DC1-3. Prior to DC4, half the rats received vehicle and half received 3 mg/kg CNO. The next day, prior to DC5, rats received the opposite drug treatment. **B** Freezing behavior during reward, fear, fear + safety, and safety cues across DC1-3 under vehicle conditions in virus-free rats. Freezing was significantly higher to the fear cue compared to the fear + safety cue during DC2 and DC3, indicating good fear discrimination (***p < 0.001, ****p < 0.0001 compared to fear cue). Freezing behavior during DC4 and DC5 also showed significantly higher freezing during the fear cue compared to the fear + safety cue under both vehicle and CNO conditions, indicating no effects on fear discrimination in virus-free rats under CNO conditions (**p < 0.01, ***p < 0.001 compared to fear cue). **C** Port behavior during reward, fear, fear + safety, and safety cues across DC1-3 under vehicle conditions in virus-free rats. Port behavior was significantly higher than all other cues across DC1-3, indicating good reward discrimination. Port behavior during DC4 and DC5 also showed significantly higher port time during the reward cue compared to all other cues under both vehicle and CNO conditions, indicating no effects on reward discrimination in virus-free rats under CNO conditions. **p < 0.01, ****p < 0.0001 compared to all other cues within session, within drug treatment.

**Fig. 2:**
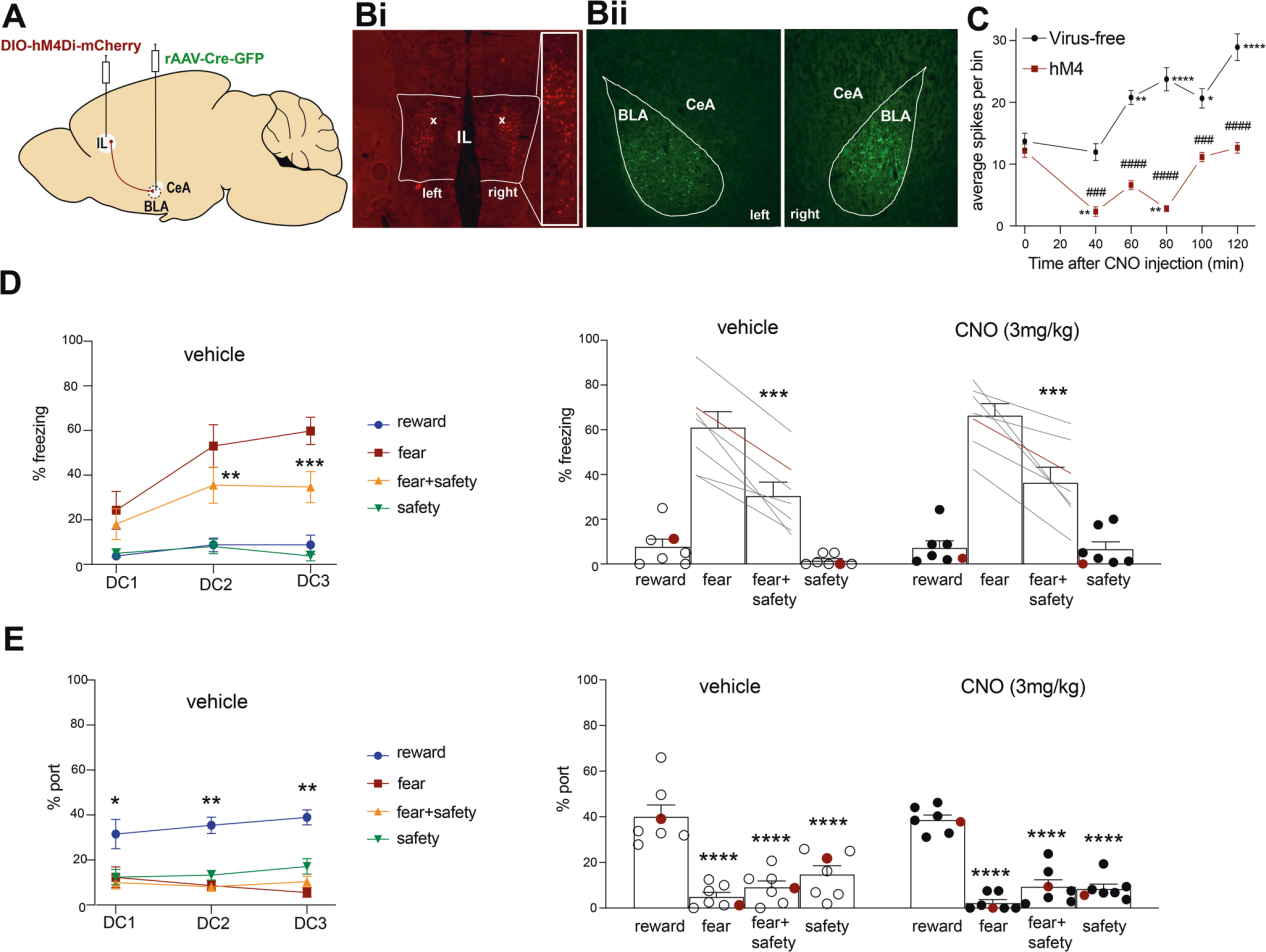
Experiment 2: chemogenetic inhibition of BLA-projecting IL neurons did not affect safety expression. **A** Schematic showing intersectional viral strategy for expressing hM4Di-mCherry in BLA-projecting IL neurons. **B**i mCherry expression for one rat in the IL with little to no spillover into prelimbic cortex. White x’s indicate the recording location from (**C**). **B**ii GFP expression for one rat in the BLA marking the infusion site, showing little to now spillover into the CeA. **C** Virus-free (*n* = 3) and hM4Di-mCherry expressing (*n* = 2) rats were taken for anesthetized IL recordings under CNO conditions. CNO injection resulted in significant inhibition of spikes per bin in hM4 rats at 40 and 60 min post-CNO injection (**p < 0.01 compared to time 0), and a significant excitation in virus-free rats at 60–120 min (*p < 0.05, **p < 0.01, ****p < 0.0001 compared to time 0). Spikes per bin was significantly lower in hM4 rats at 40–120 min compared to virus-free rats (^###^*p* < 0.001, ^####^*p* < 0.0001). **D**, left All rats received vehicle prior to DC1-3. During DC2-3, percent time spent freezing was higher to the fear cue compared to the fear+safety cue (**p < 0.01, ***p < 0.001 compared to fear cue). **D**, right Using a within-subjects design, rats received CNO before either DC4 or DC5, and vehicle before the other session. Under both vehicle and CNO conditions, percent time freezing was significantly higher to the fear cue compared to the fear + safety cue (***p < 0.001 compared to fear cue). **E**, left All rats received vehicle prior to DC1-3. For all sessions, percent time at port was higher to the reward cue compared to all other cues (*p < 0.05, **p < 0.01 compared to reward cue). **E**, right Using a within-subjects design, rats received CNO before either DC4 or DC5, and vehicle before the other session. Under both vehicle and CNO conditions, percent time at port was significantly higher to the reward cue compared to all other cues (****p < 0.0001 compared to reward cue). Data points in red from right panels of (**D**) and (**E**) are from the rat that was taken to contribute to recordings shown in (**C**).

All animals across Experiments 1–3 underwent the same behavioral training procedure. Experiment 1 consisted of virus-free rats (*n* = 16), Experiment 2 included rats with histologically verified hM4Di expression in IL -> BLA (*n* = 8), and Experiment 3 included rats with histologically verified hM4Di expression in IL -> CeA (*n* = 5), as well as histologically verified “misses” where hM4Di was not expressed (*n* = 6). Animals first received five sessions of reward training distributed across 5 days (Fig. 1A). Each session consisted of 25 pairings (ITI, 90–130 s) of the reward cue with a 3 s delivery of 10% liquid sucrose (100 μl pseudorandomly presented 10–20 s after reward cue onset) into a port (Fig. 1A; tone A + sucrose (reward)). Animals then received one session of habituation training, which consisted of 25 trials of the reward cue paired with liquid sucrose (100 μl pseudorandomly presented 10–20 s after reward cue onset) (Fig. 1A; tone A + sucrose (reward)), 5 trials of the future fear cue presented alone (Fig. 1A; tone B), and 5 trials of the future safety cue presented alone (Fig. 1A; light) (ITI, 90–130 s). This habituation procedure has been used in this task to assess and reduce any baseline freezing that may be present to the novel cues with the number of trials presented not being sufficient to produce latent inhibition [9]. Animals then received five sessions of discriminative conditioning (DC1-5) across 5 days; i.e., 1 session per day. Each session consisted of the reward cue paired with liquid sucrose (100 μl pseudorandomly presented 10–20 s after reward cue onset) (Fig. 1A; tone A + sucrose (reward)), the fear cue paired with footshock (0.5 s, 0.5 mA at cue offset) (Fig. 1A; tone B + footshock (fear)), the safety cue and fear cue presented concurrently without footshock (Fig. 1A; tone B + light (fear + safety)), and the safety cue presented alone without footshock (Fig. 1A; light (safety)). For all DC sessions the first cue was presented 12 min into the session. Sessions DC1-3 were preceded by vehicle injections 20 min prior to the session and consisted of 15 reward trials, 4 fear trials, 15 fear + safety trials, and 10 safety-alone trials (44 trials, ITI 60–120 s). For sessions DC4 and DC5, rats received either vehicle or 3 mg/kg CNO injections 20 min prior to the start of DC4, and the opposite drug treatment prior to DC5. DC4 and 5 consisted of 7 reward trials, 2 fear trials, 7 fear + safety trials, and 6 safety-alone trials (22 trials delivered apx 32–81 min post-CNO injection) in order to have all cues presented within 30–80 min post-CNO injection, the peak of CNO-induced inhibition of firing (Fig. 2C).

### Behavioral analyses

Fear behavior was assessed manually offline from videos by measuring freezing, defined as complete immobility with the exception of respiratory movement, which is an innate defensive behavior [17, 18]. The amount of time spent freezing within a 20 s interval during cue presentation was quantified and expressed as percentages. Reward behavior was assessed manually by quantifying the amount of time the animals spent inside the port or having their nose positioned at the port entrance, and was expressed as percentages. Individuals performing the manual behavioral scoring had Pearson’s correlations of at least *r* = 0.8 with other scorers in the same laboratory for freezing and reward behaviors. The behavioral data were analyzed with two-way repeated-measures ANOVAs with post hoc Dunnett’s or Sidak’s multiple comparisons in GraphPad Prism. Freezing to the fear cue was compared (Sidak’s) to the fear + safety cue and reward seeking to the reward cue was compared (Dunnett’s) to each other cue.

### Histology

Rats were included if mCherry in the IL and GFP in the amygdala target, either BLA (Experiment 2: *n* = 7) or CeA (Experiment 3: *n* = 5), were observed either unilaterally or bilaterally. Since there was a behavioral effect under CNO in the IL -> CeA group, “misses” were included as an additional control; these six rats had no apparent mCherry expression (Experiment 3). Rats with significant spillover of mCherry expression in the prelimbic cortex were excluded.

## Results

### Experiment 1: CNO in virus-free rats did not affect freezing or port behavior

All rats (*n* = 16) received reward and habituation training prior to discriminative conditioning. Data for all discriminative conditioning sessions are shown (Fig. 1B, C). Vehicle was administered i.p. 20 min prior to DC1-3. A two-way repeated-measures ANOVA of freezing behavior across DC1-3 showed a significant session X cue interaction (*F*(2,14) = 8.5, *p* = 0.004), and significant main effects of session (*F*(2,14) = 17.42, *p* = 0.0002) and cue (*F*(1,7) = 33.22, *p* = 0.0007) (Fig. 1B, left). Post hoc Sidak’s test to the fear cue showed that freezing to the fear cue was significantly higher than the fear + safety cue during DC2 and DC3, indicating good fear discrimination (DC2: fear > fear + safety (*p* < 0.0001); DC3: fear > fear + safety (*p* = 0.0005). Similarly, a two-way repeated-measures ANOVA of port behavior during the reward, fear, fear + safety, and safety cues across DC1-3 showed main effects of session (*F*(1.7, 11.58) = 4.74, *p* = 0.036) and cue (*F*(1.72, 12.04) = 106.4, *p* < 0.0001) (Fig. 1C, left). Post hoc Dunnett’s multiple comparisons test to the reward cue showed that port seeking during the reward cue was significantly higher than all other cues across DC1-3, indicating good reward discrimination (DC1: reward > fear, fear + safety, safety (all *p* < 0.0001); DC2: reward > fear (*p* = 0.0001), fear + safety (*p* = 0.001), safety (*p* = 0.0018); DC3: reward > fear (*p* = 0.0005), fear + safety (*p* = 0.0005), safety (*p* = 0.002)).

Then, following a within subjects design, half the rats received vehicle injection prior to DC4, while the other half received CNO. The next day, the drug order was reversed prior to DC5. Data were pooled together for vehicle and CNO sessions. A two-way repeated-measures ANOVA of freezing behavior showed a main effect of cue (*F*(1,15) = 22.8, *p* = 0.0002) (Fig. 1B, right). Post hoc Sidak’s test to the fear cue showed significantly higher freezing during the fear cue compared to the fear + safety cue for both vehicle (*p* = 0.0008) and CNO conditions (*p* = 0.002), indicating no significant alteration in freezing behavior under these conditions. Similarly, a two-way repeated-measures ANOVA of port behavior also showed a main effect of cue (*F*(3.45) = 69.99, *p* < 0.0001) (Fig. 1C, right). Post hoc Dunnett’s multiple comparisons test to the reward cue showed that port seeking during the reward cue was significantly higher than all other cues under both vehicle and CNO conditions (*p* < 0.0001 for all comparisons), indicating no significant alteration in port behavior under these conditions.

### Experiment 2: chemogenetic inhibition of BLA-projecting IL neurons did not affect safety expression

BLA-projecting IL neurons were targeted via an intersectional viral approach (Fig. 2A). Histological verification of mCherry in the IL (Fig. 2Bi) and GFP in the BLA (Fig. 2Bii) allowed ruling out spillage into the CeA and PL in seven rats. One of these rats was taken for electrophysiological assessment after completing the DC behavior task. An additional rat did not receive behavioral training but was taken at the time point of DC4 post-surgery for electrophysiological assessment. Three virus-free naïve rats were also taken for electrophysiological assessment. These five rats underwent anesthetized single-unit IL recordings of spontaneous activity with 3 mg/kg CNO on board. Our data indicate that the optimal inhibition window induced by CNO was ~40–80 min post-injection based on a two-way ANOVA showing a significant group × time interaction (*F*(5,687) = 9.69, *p* < 0.0001) and main effects of group (*F*(1,687) = 187.1, *p* < 0.0001) and time from injection (*F*(5,687) = 17.02, *p* < 0.0001). Sidak’s multiple comparisons test showed significant inhibition of spikes per trial (averaged spontaneous activity for a 0.5 s bin of time across 22 consecutive bins) at 40 min (*p* = 0.003) and 80 min (*p* = 0.007) post-CNO injection. Spikes per trial were also significantly lower in the hM4 group compared to virus-free naïve rats at 40, 60, 80, 100, and 120 min post-CNO injection (*p* = 0.0005, *p* < 0.0001, *p* < 0.0001, *p* = 0.0007, *p* < 0.0001, respectively). All rats in the remainder of this study had behavioral assessments limited to a time window of 30–80 min post-injection, the peak of inhibition. Also of note were the spikes per trial significantly increasing in virus-free naïve rats 60–120 min post-CNO injection (*p* = 0.009, *p* < 0.0001, *p* = 0.01, *p* < 0.0001, respectively).

As before, all rats received reward and habituation training prior to discriminative conditioning. Data for all discriminative conditioning sessions are shown (Fig. 2D, E). Vehicle was administered i.p. 20 min prior to DC1-3. A two-way repeated-measures ANOVA of freezing behavior across DC1-3 showed a significant session × cue interaction (*F*(2,12) = 4.89, *p* = 0.03), and significant main effects of session (*F*(2,12) = 15.54, *p* = 0.0005) and cue (*F*(1,6) = 127.8, *p* < 0.0001) (Fig. 2D, left). Post hoc Sidak’s test to the fear cue showed that freezing to the fear cue was significantly higher than the fear + safety cue during DC2 and DC3, indicating good fear discrimination (DC2: fear > fear + safety (*p* = 0.005); DC3: fear > fear + safety (*p* = 0.0002)). Similarly, a two-way repeated-measures ANOVA of port behavior during the reward, fear, fear + safety, and safety cues across DC1-3 showed a main effect of cue (*F*(2.08, 12.48) = 106.7, *p* < 0.0001) (Fig. 2E, left). Post hoc Dunnett’s multiple comparisons test to the reward cue showed that port seeking during the reward cue was significantly higher than all other cues across DC1-3, indicating good reward discrimination (DC1: reward > fear (*p* = 0.008), fear + safety (*p* = 0.008), safety (*p* = 0017); DC2: reward > fear (*p* = 0.0009), fear + safety (*p* = 0.0003), safety (*p* = 0.002); DC3: reward > fear (*p* = 0.0002), fear + safety (*p* < 0.0001), safety (*p* = 0.004).

Then, following a within subjects design, half the rats received vehicle injection prior to DC4, while the other half received CNO. The next day, the drug order was reversed prior to DC5. Data were pooled together for vehicle and CNO sessions. A two-way repeated-measures ANOVA of freezing behavior showed a main effect of cue (*F*(1,6) = 34.59, *p* = 0.001) (Fig. 2D, right). Post hoc Sidak’s test to the fear cue showed significantly higher freezing during the fear cue compared to the fear + safety cue under both vehicle and CNO conditions, indicating no significant effect on freezing by inhibiting BLA-projecting IL neurons (Vehicle: fear > fear + safety (*p* = 0.0001); CNO: fear > fear + safety (*p* = 0.0001)). Similarly, a two-way repeated-measures ANOVA of port behavior showed a main effect of cue (*F*(2.41, 14.48) = 89.02, *p* < 0.0001) (Fig. 2E, right). Post hoc Dunnett’s multiple comparisons test to the reward cue showed that port seeking during the reward cue was significantly higher than all other cues under both vehicle and CNO conditions, again indicating no significant effect on port behavior by inhibiting BLA-projecting IL neurons (Vehicle: reward > fear (*p* = 0.0006), fear + safety (*p* = 0.0009), safety (*p* = 0.0007); CNO: reward > fear, fear + safety, safety (*p* < 0.0001 all comparisons). Data point shown in red is the rat who underwent electrophysiological assessment after the completion of the DC task (Fig. 2C).

### Experiment 3: chemogenetic inhibition of CeA-projecting IL neurons impaired safety expression

**Fig. 3:**
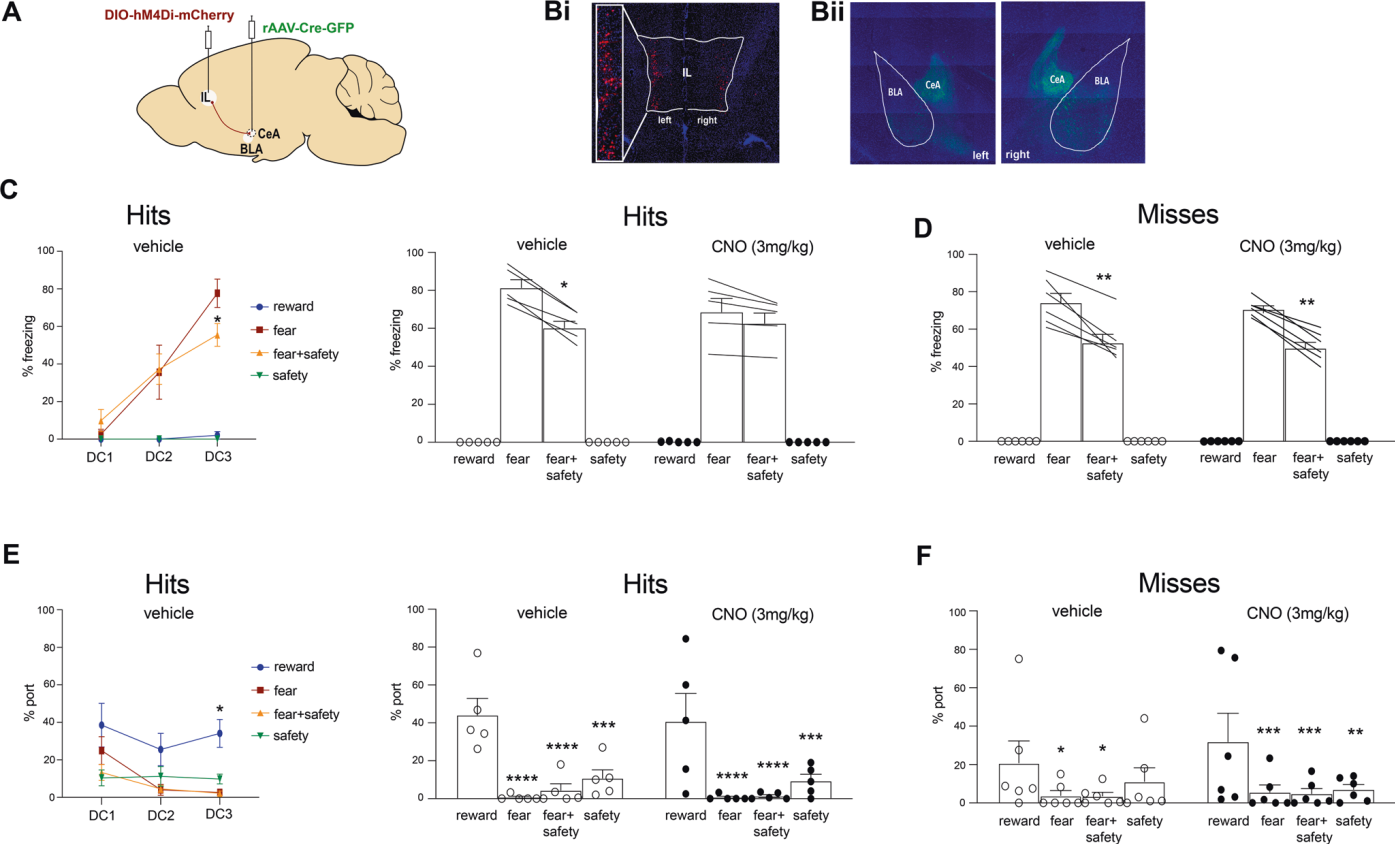
Experiment 3: chemogenetic inhibition of CeA-projecting IL neurons impaired safety expression. **A** Schematic showing intersectional viral strategy for expressing hM4Di-mCherry in CeA-projecting IL neurons. **B**i mCherry expression for one rat in the IL with little to no spillover into prelimbic cortex. **B**ii GFP expression for one rat in the CeA marking the infusion site, showing little to now spillover into the BLA. **C**, left All rats received vehicle prior to DC1-3. During DC3, percent time spent freezing was higher to the fear cue compared to the fear + safety cue (*p < 0.05 compared to fear cue). **C**, right Using a within-subjects design, rats received CNO before either DC4 or DC5, and vehicle before the other session. Under vehicle conditions, percent time freezing to the fear cue was significantly higher than the fear + safety cue but not in the CNO condition (*p < 0.05 compared to fear cue). **D** Percent time freezing in rats that either did not show detectable mCherry expression or off-target expression shown as “Misses”. Under both vehicle and CNO conditions, percent time freezing was significantly higher to the fear cue compared to the fear + safety cue (**p < 0.01 compared to fear cue). **E**, left All rats received vehicle prior to DC1-3. During DC3, percent time at port was higher to the reward cue compared to fear (*p < 0.05) and fear + safety (*p < 0.05) cues. **E**, right Using a within-subjects design, rats received CNO before either DC4 or DC5, and vehicle before the other session. Under both vehicle and CNO conditions, percent time at port was significantly higher to the reward cue than all other cues (***p < 0.001, ****p < 0.0001 compared to reward cue). **F** Percent time at port in rats that either did not show detectable mCherry expression or off-target expression shown as “Misses”. Under both vehicle and CNO conditions, percent time at port was significantly higher to the reward cue compared to all other cues (*p < 0.05, **p < 0.01, ***p < 0.001 compared to reward cue).

CeA-projecting IL neurons were targeted via an intersectional viral approach (Fig. 3A). Histological verification of mCherry in the IL (Fig. 3Bi) and GFP in the CeA (Fig. 3Bii) allowed ruling out spillage into the BLA and PL in five rats.

As before, all rats received reward and habituation training prior to discriminative conditioning. Data for all discriminative conditioning sessions are shown (Fig. 3C–F). Vehicle was administered i.p. 20 min prior to DC1-3. A two-way repeated measures ANOVA of freezing behavior across DC1-3 showed a significant session × cue interaction (*F*(2,8) = 4.5, *p* = 0.04), and main effect of session (*F*(2,8) = 29.28, *p* = 0.0002) (Fig. 3C, left). Post hoc Sidak’s test to the fear cue showed that freezing to the fear cue was significantly higher than the fear + safety cue during DC3, indicating good fear discrimination by DC3 (fear > fear + safety (*p* = 0.04)). Similarly, a two-way repeated measures ANOVA of port behavior during the reward, fear, fear + safety, and safety cues across DC1-3 showed a main effect of cue (*F*(1.26, 5.05) = 14.15, *p* = 0.011) (Fig. 3E, left). Post hoc Dunnett’s multiple comparisons test to the reward cue showed that port seeking during the reward cue was significantly higher than the fear (*p* = 0.046) and fear + safety (*p* = 0.035) cues during DC3, indicating good reward discrimination by DC3.

Then, once again following a within subjects design, half the rats received vehicle injection prior to DC4, while the other half received CNO. The next day, the drug order was reversed prior to DC5. Data were pooled together for vehicle and CNO sessions. A two-way repeated measures ANOVA of freezing behavior showed a main effect of cue (*F*(1, 4) = 9.31, *p* = 0.038) (Fig. 3C, right). Post hoc Sidak’s test to the fear cue showed significantly higher freezing during the fear cue compared to the fear + safety cue under only vehicle conditions (fear > fear + safety (*p* = 0.04)). Under CNO conditions, freezing to the fear cue was not significantly higher than the fear + safety cue (*p* = 0.65) (Fig. 3C, right), indicating the inhibition of CeA-projecting IL neurons impaired the downregulation of fear in the presence of the safety cue; i.e., safety expression. Port behavior, on the other hand, was not affected. A two-way repeated-measures ANOVA of port behavior showed a main effect of cue (*F*(3,12) = 11.67, *p* = 0.0007) (Fig. 3E, right). Post hoc Dunnett’s multiple comparisons test to the reward cue showed that port seeking during the reward cue was significantly higher than all other cues under both vehicle and CNO conditions, indicating no significant effect on port behavior by inhibiting CeA-projecting IL neurons (Vehicle: reward > fear (*p* < 0.0001), fear + safety (*p* < 0.0001), safety (*p* = 0.0002); CNO: reward > fear (*p* < 0.0001), fear + safety (*p* < 0.0001), safety (*p* = 0.0003).

Even though we demonstrated that CNO had no effect on safety expression in naïve virus-free rats (Fig. 1), we also assessed rats that were not histologically classified “hits”, but instead “misses” (*n* = 6), and exposed to the same surgical procedures and CNO exposure as those classified as “hits”. This included rats with no apparent expression. Here, both freezing to the fear cue (main effect of cue (*F*(1,15) = 127.3, *p* < 0.0001)) (Fig. 3D) and port behavior to the reward cue (main effect of cue (*F*(3,15) = 4.13, *p* = 0.03)) (Fig. 3F) were significantly higher than any other cue, under both vehicle and CNO conditions. (Freezing, Vehicle to fear > fear + safety (*p* = 0.007); Freezing, CNO to fear > fear + safety (*p* = 0.009); Port, Vehicle to reward > fear (*p* = 0.02), fear + safety (*p* = 0.02); Port, CNO to reward > fear (*p* = 0.0009), fear + safety (*p* = 0.0007), safety (*p* = 0.001)). This further supports the lack of freezing suppression during the fear + safety cue in those classified as “hits” was not simply due to non-specific effects of CNO.

### Fear suppression ratios to quantify levels of fear suppression in the presence of the safety cue

**Fig. 4:**
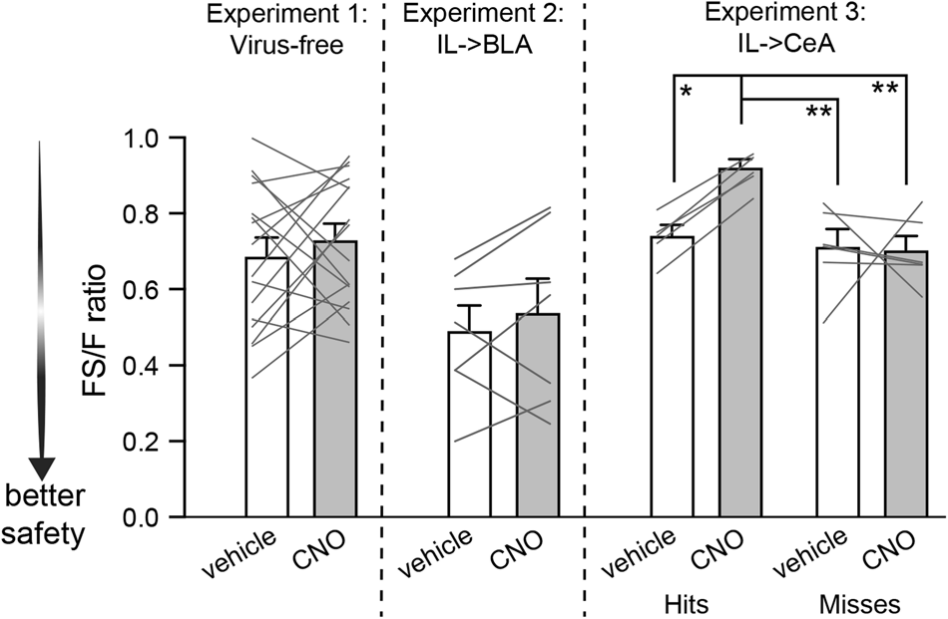
Fear suppression ratios across experiments. For each rat within each experiment, the amount of freezing to the fear + safety cue (FS) was divided by freezing to the fear cue (F) to create a FS/F ratio. The lower the ratio, the more freezing was suppressed to the FS cue compared to the F cue; i.e., better safety. Ratios are shown for each within-subject experiment with each line representing one rat tested under vehicle and CNO conditions. All rats classified as IL -> CeA “Hits” showed worse suppression ratios under CNO conditions compared to vehicle conditions (*p < 0.05), as well as compared to “Misses” under either CNO (**p < 0.01) or vehicle (**p < 0.01) conditions.

To better appreciate the within-subjects design as well as quantify the level of fear suppression during the fear + safety cue compared to the fear cue of each subject under the various conditions of this study, we calculated fear suppression ratios by taking the amount of freezing to the fear + safety cue (FS) and dividing it by the amount of freezing to the fear cue (F) for each individual rat (Fig. 4). The lower the FS/F ratio, the more substantial suppression of fear to the fear + safety cue, i.e., better safety. Data shown are linked within animal to show these ratios within-subject under vehicle and CNO conditions. Data for each experiment were analyzed separately due to the unbalanced group numbers and group types, as well as due to some of the variability in fear suppression under control conditions across the three experiments. The fear suppression ratios were not significantly different between vehicle and CNO conditions in the virus-free rats (Experiment 1; paired *t*-test, *p* = 0.45) or the rats expressing hM4Di in IL -> BLA neurons (Experiment 2; paired *t*-test, *p* = 0.44), indicating safety expression was equivalent under vehicle and CNO. However, in rats expressing hM4Di in IL -> CeA (Hits; Experiment 3), fear suppression ratios were significantly higher under CNO compared to vehicle within animal (one-way ANOVA, *F*(3,18) = 7.7, *p* = 0.002), indicating better safety expression under vehicle (*p* = 0.02). Moreover, fear suppression ratios for these rats under CNO were also significantly higher than rats classified as “Misses” and tested under CNO (*p* = 0.002) or vehicle (*p* = 0.004).

## Discussion

Using an intersectional virus approach to restrict expression of hM4Di to IL neurons projecting to either the BLA or CeA, we showed CeA-projecting IL neurons were necessary for safety expression, and that BLA-projecting IL neurons were not. That is, inhibiting IL -> CeA neurons prevented the typical reduction in freezing to the fear cue in the presence of a safety cue (fear + safety cue). In our prior work we showed that inhibiting IL activity with muscimol and baclofen resulted in very similar behavioral effects: freezing to the fear and fear + safety cues were equivalent [7]. The work presented here implies these effects in our prior work were most likely driven by inhibiting the IL -> CeA neurons.

IL signaling has been implicated in situations where an adaptive behavior needs to be selected over a maladaptive behavior in times of conflicting or competing circumstances [10]. For example, many have shown a critical role of IL in fear extinction recall [7, 19–21], a time point where the extinguished cue may appear ambiguous depending on time since or location where extinction training occurred. Do-Monte et al. [13] optogenetically increased or decreased IL input to the BLA during cued fear extinction training, and found no observable effects on the extinction acquisition curve itself, but later extinction recall (stimulation-free) was bidirectionally affected: increased IL input resulted in better extinction recall and, conversely, decreased IL input resulted in less extinction recall. Meanwhile, when IL input to the BLA was inhibited instead during extinction recall, it had no effect, indicating IL’s input to BLA was driving consolidation of fear extinction memory and not the expression or recall of learned fear extinction. Similarly, Bloodgood et al. [22] have shown chemogenetic inhibition of BLA-projecting IL neurons during fear extinction training did not affect within session reduction of freezing, but did impair the recall of fear extinction the next day under drug-free conditions. Our data presented here are consistent with these extinction data. That is, IL signaling to BLA may be more critical during acquisition of fear extinction and, possibly, discriminative safety compared to later time points, after the behavior has been learned. We showed here, that once learned, expression of learned fear suppression to a safety cue does not depend upon IL signaling to the basolateral amygdala, but does depend upon IL signaling to the central amygdala.

Fear suppression as a result of extinction training has repeatedly been shown to be context-specific (reviewed in [23]). Our study did not investigate whether or not suppressed fear to the fear + safety cue would be observable in contexts other than the training context. At a behavioral level, we would predict the fear suppressing ability of the safety cue would transfer to a new context, given all rats had reward training before the DC sessions in the same context, and that we have not observed any background freezing or reward seeking in any of our prior studies using the same paradigm [4–7]. However, whether or not the same manipulations of IL -> BLA or IL -> CeA would have the same effect in a new context is unclear and remains to be tested.

In other safety conditioning studies, Falls and Davis [24] have previously investigated the role of the central amygdala in conditioned inhibition of a fear potentiated startle response by lesioning the CeA after training to a safety cue. Unlike our study, which used compound fear + safety cues, Falls and Davis used a serial presentation design in which the safety cue was followed by the fear cue, without shock delivery. Their post-lesion test showed a lack of fear potentiated startle response expression to all cues, including fear cues, making it unclear whether or not the CeA was needed for the recall of safety. When those same animals were retrained afterwards, all animals showed an increase startle to the fear cue and a significant reduction when the safety cue was presented, although it was not as pronounced as before surgery or compared to sham controls. That is, the CeA was not needed for safety acquisition and is in line with our data implicating the CeA in expressing already learned safety.

In our prior work, where a fear cue conflicts with a safety cue (fear + safety cue), the IL was necessary to inhibit fear during this compound cue [7]. We have also recently reported that the IL contains a large proportion of neurons with excitatory responses to the fear + safety cue specifically [3]. We also observed a separate group of neurons with excitatory responses to both the fear + safety and reward cues, as well as bidirectional neurons with excitation to the fear + safety cue and inhibition to the fear cue [3]. In most cases, IL neurons responded to the fear + safety cue but not to the safety cue when presented alone. This is in contrast to our other previous work showing safety-related activity in the basal amygdala (BA), where significant changes in firing rates were seen consistently to both the safety cue and fear + safety cue [9]. We have yet to record in the central amygdala, but it is tempting to speculate that cue-evoked responding may appear more similar to what we observed in the IL; that is, selective changes in firing rates to the fear + safety cue, but not the safety cue alone. The safety-related responses we observed in both the BA and IL developed over sessions indicating they were learned responses. However, we have not been able to tease apart the timeline of how these safety-related neural responses develop against the development of safety behavior. We hypothesize that the IL may first be receiving increased input from the BLA, and then IL increases its output to the BLA, during safety acquisition since reciprocal projections between the BLA and IL have been shown to be important in the acquisition and consolidation of fear extinction [19]. Once acquired though we hypothesize that IL’s input to the CeA is what is critical for effective safety expression. Interestingly, Lay et al. [25] have recently shown in an appetitive extinction task that neuronal ensembles within the CeA were critical for extinction retrieval, while those in the BLA were not, thus fitting in our proposed BLA vs. CeA framework.

The question remains as to which neurons within the CeA are receiving the IL input to affect safety behavior expression. Single-unit recordings in the lateral region of the CeA (CeA-L) during a fear conditioning task have shown that there are separate microcircuits of GABAergic cells within this region that are either excited (“ON”) or inhibited (“OFF”) by a fear cue, and that these microcircuits inhibit each other [26]. This implies the CeA-L neurons that are inhibited by a fear cue may be regulating safety behavior, especially if the fear cue is in conflict with a safety cue, which could be mediated by the “ON” and “OFF” circuits inhibiting each other. These “OFF” cells that are inhibited by a fear cue are thought to be PKCδ+ [27], and PKCδ+ neurons within CeA-L have been shown to be required to form a fear extinction memory [28]. Based on this, we hypothesize that the PKCδ+ cells in the CeA-L may be receiving increased input from the IL after a safety cue has been learned to drive safety behavior under conflict (i.e., the fear + safety cue), which ultimately would be seen as decreased output from the medial region of the CeA and decreased freezing behavior.

We have yet to test the role of BLA- or CeA-projecting IL neurons in safety acquisition, but we expect that BLA-projecting IL neurons would be necessary to acquire safety in our task. If so, it would be interesting to increase activity from IL to BLA during training to improve safety acquisition. Our prior work has shown that female Long Evans rats do not significantly suppress freezing to the fear + safety cue compared to the fear cue [5]. In fact, the females in this task looked very similar to the males in the current study under IL -> CeA chemogenetic inhibition. Thus it would be particularly interesting to test if increased IL -> BLA signaling during safety acquisition could overcome this safety-deficit in females. However, using a chemogenetic approach with CNO to test this should be used with caution as our recording data in anesthetized virus-free rats showed a gradual increase in neural activity within the IL under CNO conditions (Fig. 2C). This would have presumably increased output to all IL targets. However, our virus-free behavioral data with CNO did not seem to improve safety expression in any observable way (Figs. 1B and 4), at least not with a single dose during recall. Whether or not CNO on its own could potentially improve safety acquisition if given throughout training would need to be carefully assessed in any future study assessing safety acquisition. Additionally, it should be noted that our recordings did not include a virus-free vehicle injection, leaving open the possibility that a vehicle injection could potentially gradually increase spontaneous firing in the IL under anesthesia independent of CNO.

The loss of discriminative fear regulation that we observed here during IL -> CeA inhibition is similar to the behavioral disruption seen in post-traumatic stress disorder (PTSD) individuals that fail to regulate fear adaptively when safety cues coincide with fear cues [2]. Since a loss of appropriate discriminative fear regulation can result in generalized and persistent fear responses to nonthreatening stimuli [1], it is important to tease apart the behavioral and circuit mechanisms of how to effectively dampen fear under safe conditions. Our data advances our understanding of the neural underpinnings in restoring compromised behavioral control over fear under safety/threat conflict.
